# Effect of LED Spectrum on the Quality and Nitrogen Metabolism of Lettuce Under Recycled Hydroponics

**DOI:** 10.3389/fpls.2021.678197

**Published:** 2021-06-17

**Authors:** Jie Li, Tao Wu, Ke Huang, Yubing Liu, Mingyue Liu, Junwei Wang

**Affiliations:** ^1^College of Horticulture, Hunan Agricultural University, Changsha, China; ^2^Engineering Research Center for Horticultural Crop Germplasm Creation and New Variety Breeding, Ministry of Education, Changsha, China; ^3^Key Laboratory for Vegetable Biology of Hunan Province, Changsha, China

**Keywords:** lettuce, LED, quality, nitrogen reduction, nitrogen assimilation

## Abstract

Light quality optimization is an efficient method for improving the growth and quality of lettuce in plant factories. In this study, lettuce seedlings were illuminated under different light-emitting diode (LED) lights, namely, red-blue (RB), red-blue-green (RBG), red-blue-purple (RBP), and red-blue-far-red (RBF) LED lights, to investigate the effect of light quality on growth, quality, and nitrogen metabolism. The combination of 75% red and 25% blue light was set as the basic light source, and 20% of green, purple and far-red light were added to basic light source, respectively. All the treatments were set to 200 μmol m^–2^ s^–1^. Results showed that the fresh weight and dry weight of aboveground lettuce under RBG, RBP, and RBF treatments were significantly lower than those under the RB treatment because of the decrease in the effective photon flux density for chlorophyll absorption. The vitamin C content of the lettuce leaves was increased by about 23% with the addition of purple light. For nitrate reduction, the addition of green light significantly increased the nitrite content of the lettuce leaves. It also promoted the reduction from nitrite to ammonium through the activation of the nitrite reductase (*NiR*) expression and enzyme activity. The nitrate and ammonium content decreased with the addition of purple light because of the inhibited *NR* and *NiR* expression and enzyme activity. For nitrogen assimilation, individual (e.g., Asp, Glu, and Leu) and total amino acids were induced to increase by adding green, purple, and far-red light. The addition of light was hypothesized to have inhibited protein biosynthesis, thereby causing the accumulation of amino acids. Correlation analysis showed that the relative expression levels between *HY5* and *NR*/*NiR* presented a significantly negative correlation. Transcription factor HY5 might mediate the regulation of light quality on nitrogen metabolism by inhibiting *NR* and *NiR* expressions. It might also exert a negative effect on nitrate reduction. Further studies via genome editing techniques on the identification of HY5 functions for nitrate assimilation will be valuable. Nevertheless, the results of this work enrich the understanding of the effect of light quality on nitrate metabolism at the level of gene expression and enzyme activity.

## Introduction

With the rapid development of greenhouse agriculture, plant factory production and greenhouse light supplementation have become key technologies in protected horticulture production. Hydroponics in plant factories offers numerous advantages, such as high yield, good quality, continuous production, efficient resource utilization, and avoidance of the impacts of environment change; it has been used to solve the unstable vegetable supply and the pollution of land resources (Viršilë et al., 2020; Zou et al., 2020). However, hydroponics production can cause the accumulation of nitrate nitrogen in plants, especially in leaf vegetables, such as lettuce (*Lactuca sativa L.*) (Rouphael et al., 2018). Excessive nitrate can be converted into nitrite, which can cause methemoglobinemia and carcinogenic nitrosamine in the human body (Chan, 2011). Lettuce is one of the main vegetable crops produced by hydroponics in plant factories, and it is widely consumed in its raw state because of its taste and high nutritional value. However, lettuce is a hyperaccumulator of nitrates, that is, it easily accumulates high nitrate content in its leaves (Bian et al., 2018). In accordance with the division of nitrate content in fresh vegetables, researchers have found that the nitrate content of lettuce is ≥2,500 mg/kg of its fresh weight (FW). Such content reflects the vegetable’s excessive nitrate accumulation. Therefore, an important issue is to control the nitrate content of lettuce within a reasonable range.

The physiological theory of the excessive accumulation of nitrate in vegetables can be attributed to the rate of nitrate absorption by vegetables being greater than the rate of nitrate assimilation. Nitrate is one of the most abundant nitrogen (N) sources in natural and agricultural systems, and it is the main form of nitrogen absorbed by plants (O’Brien et al., 2016). Nitrate could be reduced to ammonium through nitrate reductase (NR) and nitrite reductase (NiR). NR catalyzes the reduction of nitrate to nitrite in plants. Then nitrite is reduced to ammonium under the action of NiR. Ammonium is assimilated into organic nitrogen under the action of the glutamate synthase (GOGAT) and glutamine synthetase (GS) cycle and glutamate dehydrogenase (GDH) pathway. Several strategies can be used to reduce excessive nitrate accumulation through the regulation of enzymatic activities and gene expression-related nitrogen metabolism. Recently studies have focused on the optimization of supplemental light and the effective ways to regulate nitrate accumulation in lettuce in plant factories (Hytönen et al., 2017; Viršilë et al., 2019; He et al., 2021).

For green plants, light is an important environmental factor for life and is involved in the regulation of growth, morphology, and metabolism (Viršilë et al., 2020; Samuolienë et al., 2021). Light-emitting diodes (LEDs) are new illuminants with advantageous properties, including energy conservation, long life, small size, light weight, and high light efficiency. These advantages make LEDs a perfect light source for artificially regulating crop growth and development (Amoozgar et al., 2017; Ferreira et al., 2017). Recent studies have shown that the combination of red and blue light serves as a highly efficient light source for promoting lettuce growth (Chen et al., 2016; Amoozgar et al., 2017). The addition of certain green, purple, far-red, and UV-C light could affect the content of chemical substances in lettuce, such as nitrate, nitrite, vitamin C (Vc), amino acid content, and antioxidant capacity (Naznin et al., 2019; Riga et al., 2019; Viršilë et al., 2020; He et al., 2021). For example, the addition of green light relative to red and blue LED significantly increases NR, NiR, GOGAT, and GS activities (Bian et al., 2018). Purple light could significantly reduce nitrate accumulation and the activities of N metabolism-related enzymes, such as NR, NiR, GS, and GOGAT (Zhang et al., 2018). In sum, light quality is an important factor in the effective regulation of the growth, quality, and nitrate accumulation of lettuce in plant factories. Moreover, green, purple, and far-red light are often used as supplemental light sources and are added to basic light sources, such as white light or a combination of red and blue light.

In response to environmental changes, plants have developed a suite of photoreceptors, such as phytochromes (phy) and cryptochromes, to monitor light availability and quality. Specifically, phy are sensitive to irradiation by red and far-red light, and they uniquely function by measuring the relative quantity of each of these wavelengths (Rockwell et al., 2006). Higher plant genomes encode a suite of phytochrome proteins, including phyA, phyB, phyC, phyD, and phyE. Cryptochrome is the receptor of blue or UV-A light and encodes genes (*CRY1* and *CRY2*) in plants. Previous reports have shown that the absorption of light by different photoreceptors leads to the modulation of the core COP1/SPA complex, which further regulates downstream transcription factors to adjust plant growth and development (Gangappa and Botto, 2016; Kim et al., 2017; Roeber et al., 2021). Transcription factor HY5 (LONG HYPOCOTYL5) is a central regulator via the COP1/SPA complex, and it can be activated by photoreceptors to promote photomorphogenesis downstream to phytochromes, cryptochromes, and UV-B photoreceptors (Gangappa and Botto, 2016). However, whether HY5 mediates the regulation of light quality on the activity and expression of the NR gene in lettuce has yet to be fully studied.

According to previous research, the combination of red and blue light serves as a high-efficiency light source to promote lettuce growth. Therefore, using the combination of red and blue LEDs light as the basic light source, we investigated the effect of adding green, purple, or far-red light to basic light sources on the growth, quality, and nitrogen metabolism of lettuce. The mechanism of light quality on nitrogen metabolism was discussed on the basis of the expression level of genes related to light response and nitrogen metabolism. The results of this work should serve as a reference for supplemental light optimization in lettuce production and enable a good understanding of the effect of light quality on nitrate assimilation on the basis of the level of gene expression and enzyme activity.

## Materials and Methods

### Plant Material

Experiments were conducted in an artificial climate chamber at Hunan Agriculture University (Latitude: 27.55°N, Longitude: 113.92°E), Changsha, China, from 2 May 2019 to 28 July 2019. After soaking and germination, lettuce (*Lactuca sativa* var. cv Yidali, from Clover Seed, Co., Ltd., China) seeds were sown in rock wool (25 mm × 25 mm × 40 mm) and placed in artificial climate chamber. The climate chamber measured 4,050 mm (length) × 2,218 mm (width) × 3,000 mm (height), and comprised six three-layers shelves and a temperature, relative humidity and LED illumination control system. The growth conditions of lettuce were set as follows: white light with 12 h photoperiod (200 mmol m^–2^ s^–1^), 25°C (daytime)/18°C (night), and 75% relative humidity. When the first true leaf was unfolded, a half unit of the garden test nutrient solution (Supplementary Table 1) was used (Hori, 1966). After the second true leaf was fully unfolded, seedlings on the same site were transplanted into hydroponic tanks (1,800 mm × 650 mm × 100 mm) containing half-strength garden test nutrient solution. The nutrient solution was set as follows: EC of 1.2 dS/m, pH of 6.4, depth of 8 cm, and continuously aeration with an air pump at an interval of 20 min to maintain the dissolved oxygen at 8.0 ± 0.2 mg L^–1^. The nutrient solution was recycled every 7 days.

**FIGURE 1:**
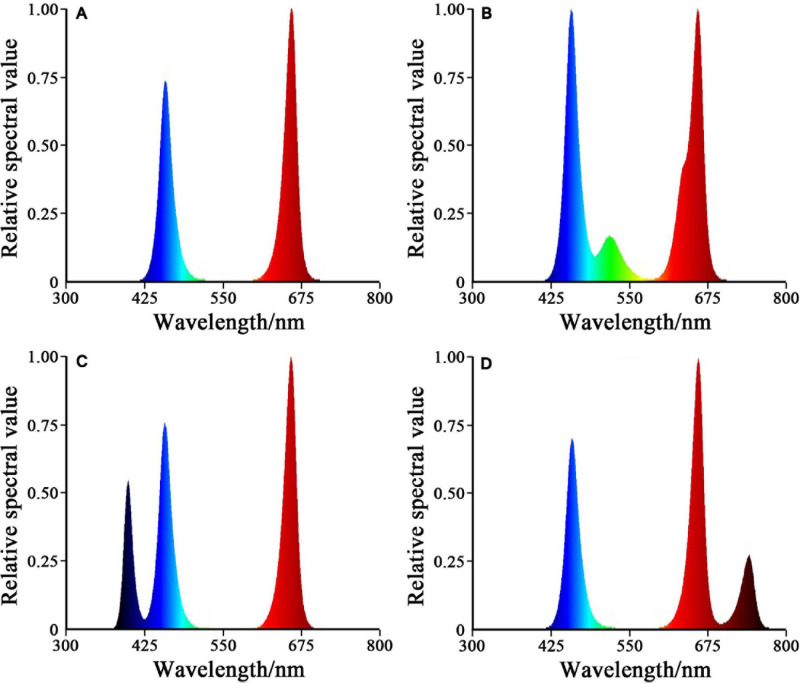
Relative spectral value of four treatments. **(A)** RB, 75% red + 25% blue LED. **(B)** RBG, 60% red + 20% blue + 20% green LED. **(C)** RBP, 60% red + 20% blue + 20% purple LED. **(D)** RBF, 60% red + 20% blue + 20% infrared LED.

After 5 days of preculture under the white LED illumination with 12 h photoperiod (200 mmol m^–2^ s^–1^), the seedlings were subjected to four experimental illumination treatments. Four light treatments were as follows: (1) red-blue (RB), 75% red + 25% blue LED light; (2) red-blue-green (RBG), 60% red + 20% blue + 20% green LED light; (3) red-blue-purple (RBP), 60% red + 20% blue + 20% purple LED light; (4) red-blue-far-red (RBF), 60% red + 20% blue + 20% infrared LED light. The light intensity of all the treatments was set to 200 ± 10 mol m^–2^ s^–1^. The LED light source (T8) was from Shanzai Agriculture and Forestry Technology Co., Ltd., Zhongshan, China. Photon fluxes were measured with a spectroradiometer (PLA-20, Everfine Optoelectronic Information Co., Ltd., Hangzhou, China). The light intensity was adjusted on the basis of the distance between the light source and the lettuce canopy. The center wavelength of each light was as follows: red light, 660 nm; blue light, 440 nm; green light, 530 nm; purple light, 410 nm; infrared light, 750 nm. The spectral values of the four treatments are shown in Figure 1.

The seedlings were organized in a complete randomized block design with three replicates per treatment, for a total of 180 seedlings in the four treatments (45 seedlings per treatment). After 25 days of transplanting, the lettuce plants under different treatments were sampled, and the related indexes were determined. The sample parts were immediately frozen in liquid nitrogen and stored at −80°C until analysis.

### Measurement of Lettuce Biomass and Chlorophyll Content of Leaves

At 25 days after treatment, the lettuce was harvested. The plants’ shoot FW and dry weight (DW) were examined using an electronic balance. Five plants were then randomly selected from each replicate, for DW deactivated at 105°C, and then dried at 75°C for 72 h.

Chlorophyll content was measured calorimetrically according to Holden’s method. Briefly, 0.2 g of fresh leaves was dipped in 10 mL of a mixture of acetone, ethanol, and water (4.5:4.5:1, v/v/v) until they turned white to extract the chlorophyll a and b content. The absorbance of the extract liquor was determined using an ultraviolet spectrophotometer at 645 nm (OD_645_) and 663 nm (OD_663_). The chlorophyll content was calculated as follows: chlorophyll a content (mg/g) = 12.7 × OD_663_ − 2.69 × OD_645_; chlorophyll b content (mg/g) = 22.9 × OD_645_ − 4.86 × OD_663_

### Measurement of Lettuce Quality

The contents of soluble sugar, soluble protein, and Vc of the lettuce leaves were determined using kits (Shanghai ZCIBIO Technology Co., Ltd., Shanghai, China). The measurement of soluble sugar was based the Anthrone method (Liu et al., 2011). Exactly 0.1 g of samples was mixed with 1 mL distilled water and ground into a homogenate, soaked in a 95°C water bath for 10 min, and then centrifuged at 25°C for 10 min. The supernatant was diluted to 10 mL (distilled water). Soluble sugar was extracted with the prepared solution in the kit, and the absorbance was measured at 620 nm. The measurement of soluble protein and Vc was based on the Coomassie brilliant blue colorimetry method (Bradford, 1976) and 2,6-dichlorophenol-indophenol method (Nielsen, 2017). The absorbance of soluble protein determined using the ultraviolet spectrophotometer was 595 nm.

### Measurement of Nitrate, Nitrite, Ammonium, and Amino Acids Contents

Fresh samples collected from the third-young, fully expanded leaves were used to determine the nitrate, nitrite, and ammonium content. Nitrate was extracted and quantified in accordance with a previously described method (Cataldo et al., 1975) with minor modifications. The principle is that nitrate reacts with salicylic acid to produce nitrosalicylic acid. The absorbance was determined to be 410 nm by using the ultraviolet spectrophotometer. Nitrite was extracted and determined in accordance with a previously described method (Stevens and Oaks, 1973). Briefly, the leaf sample was homogenized using sulfanilamide and *N*-(1-Naphthyl)-ethylene-diamine dihydrochloride. The absorbance was determined to be 550 nm by using the ultraviolet spectrophotometer. Ammonium was extracted and determined in accordance with a previously reported method (Solórzano, 1969). The principle is that ammonium reacts with ninhydrin to produce a blue compound. The absorbance was determined to be 580 nm by using the ultraviolet spectrophotometer.

Free amino acids were assayed in accordance with a previously reported method given by Shu et al. (2016). In brief, 0.1 g of dry samples was used to extract free amino acids by utilizing HCl at 110°C for 24 h. The cooled hydrolysate was filtered, dissolved, and evaporated under vacuum. The final dry matter was dissolved in citrate buffer (67 mM, pH = 2.2) and used for analysis. The content of free amino acid was determined with an automatic amino acid analyzer (Hitachi L-8900, Japan).

### Assay of Enzyme Activity of Nitrogen Metabolism

The NR activity was determined according to the method reported by Yaneva et al. (2002). Briefly, 0.5 g of fresh leaf was used to extract NR by utilizing 15 mL of phosphate (0.1 M, Ph = 7.5) at 4°C for 15 h. The supernatant was added to a reaction solution containing 200 μM KNO_3_ and 0.2 μM NADH. The amount of catalyzed production of 1 μmol NO_2_^–^ per gram of fresh sample per hour was regarded as one NR activity unit. The NiR activity was determined by the method given in the literature (Datta and Sharma, 1999). Enzyme was extracted with 50 mM phosphate buffer (containing 1 mM EDTA, 25 mM cysteine, and 3% BSA; pH = 8.8). In the reaction, 50 mM NaNO_2_ was the reaction substrate, and 5 mM methyl viologen was the electron donor. The unit of NiR activity was defined as the molar mass of NO_2_^–^ reduced per gram of leaf per minute.

The method described by Cánovas et al. (1991) was used for GS activity measurement, and the method by Singh and Srivastava (1986) was used for GOGAT activity measurement. Enzymes were extracted with 50 mM phosphate buffer (2 mM EDTA, 2 mM dithiothreitol, 1% insoluble polyvinylpyrrolidone, and 1.5% soluble casein, pH = 7.5). The supernatant was used for the GS and GOGAT activity assay. The activity of GS was expressed as μmol γ-glutamylhydroxamate formed per gram per minute. The GOGAT activity was defined as μmol NADH oxidized per gram per min. GDH was extracted using assay kits (Shanghai ZCIBIO Technology Co., Ltd., Shanghai, China), and the activity was calorimetrically measured at 340 nm by the ultraviolet spectrophotometer. The number of moles of NADH consumed per minute was defined as the unit of enzyme activity.

### RNA Extraction and Genes Expression Analysis

Total RNA was extracted from fresh lettuce (0.1 g) by using the RNA prep Pure Plant Kit: Polysaccharides and Polyphenolics-rich (Tiangen, Beijing, China) in accordance with the manufacturer’s instructions. cDNA was synthesized from 1 μg of total RNA by using an MMLV reverse transcriptase kit (Transgen, China) in accordance with the manufacturer’s instructions. For the fluorescent quantitative PCR analysis of the gene expression, the sequences of *NR*, *NiR*, *GS*, *GOGAT*, *GDH*, *phyA*, *phyB*, *phyE*, *CRY1*, and *HY5* were amplified with primers designed on the basis of the sequences obtained from NCBI (Supplementary Table 2). The expression data were analyzed with the ΔΔCt method as previously described (BioRad Real-time PCR Application guide) (Kenneth and Thomas, 2002).

### Statistical Analysis

Experimental data were processed with GraphPad Prism 5. Duncan’s multiple comparison tests at *P* < 0.05 level of significance was used to conduct one-way ANOVAs. Principal component analysis (PCA) was used to calculate the correlation between indicators and the comprehensive effect of different light conditions on the nitrogen metabolism of lettuce.

## Results and Analysis

### Effect of LED Illumination Spectra on Lettuce Biomass

**FIGURE 2:**
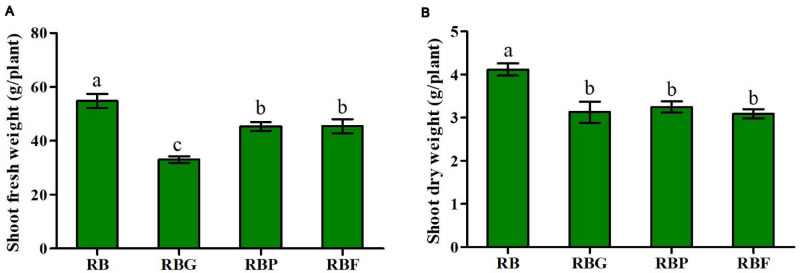
Effects of spectra on shoot fresh weight **(A)** and dry weight **(B)** in lettuce. RB, 75% red + 25% blue LED; RBG, 60% red + 20% blue + 20% green LED; RBP, 60% red + 20% blue + 20% purple LED; RBF, 60% red + 20% blue + 20% infrared LED. The letters represent the significant difference of different treatments (*p* < 0.05).

The FW and DW of aboveground lettuce under the RBG, RBP, and RBF treatments were significantly lower than those under the RB treatment (Figures 2A,B). Relative to those under the RB treatment, the FW values under the RBG, RBP, and RBF treatments decreased by 39.7, 17.2, and 17.1%, respectively. Meanwhile, the DW values under the RBG, RBP, and RBF treatments decreased by 24.0, 21.2, and 25.0%, respectively. The chlorophyll content showed an insignificant change when green, purple, or far-red light was added to the red and blue light combination. The values of chlorophyll a, b, and a + b in the lettuce leaves showed a more significant increase under the RBP and RBF treatments than under the RB treatment. Meanwhile, the values of chlorophyll a and a + b under the RBG treatment showed a more significant decrease than those under the RB treatment (Table 1).

**TABLE 1:** Effects of spectra on the quality of lettuce under different LED treatments.

**Light treatments**	**Chl a (mg/g)**	**Chl b (mg/g)**	**Chl a + b (mg/g)**	**Soluble sugar (mg/g FW)**	**Soluble protein (mg/g FW)**	**Vitamin C (mg/kg FW)**
RB	0.446 ± 0.026c	0.120 ± 0.015b	0.566 ± 0.034c	5.73 ± 0.28a	13.17 ± 0.62a	118.8 ± 8.64ab
RBG	0.319 ± 0.019d	0.120 ± 0.034b	0.439 ± 0.011d	4.53 ± 0.32b	8.84 ± 0.64bc	110.4 ± 7.59b
RBP	0.734 ± 0.006a	0.210 ± 0.028a	0.944 ± 0.021a	5.00 ± 0.12ab	10.10 ± 0.82b	146.3 ± 9.91a
RBF	0.585 ± 0.027b	0.173 ± 0.016a	0.758 ± 0.054b	4.69 ± 0.13b	6.830 ± 0.36c	104.7 ± 9.00b

*RB, 75% red + 25% blue LED; RBG, 60% red + 20% blue + 20% green LED; RBP, 60% red + 20% blue + 20% purple LED; RBF, 60% red + 20% blue + 20% infrared LED.*

*The letters represent the significant difference of different treatments (*p* < 0.05).*

### Effect of LED Illumination Spectra on Lettuce Quality

The soluble sugar content of lettuce leaves under the RBG and RBF treatments was significantly lower than that under the RB treatment, indicating a decrease of 20.9 and 18.2%, respectively (Table 1). The soluble protein content of lettuce leaves decreased more significant under RBG, RBP, and RBF light than that under the RB treatment. The percentage of decreased value was, respectively, 32.9, 23.3, and 48.1%. Relative to that under RB light, the Vc content of lettuce leaves under RBP light increased by about 23%. It decreased slightly under the RBG and RBF conditions relative to that in the RB treatment, but it did not reach the significance level.

### Effect of LED Illumination Spectra on Nitrogen Reduction and Assimilation in Lettuce Leaves

**FIGURE 3:**
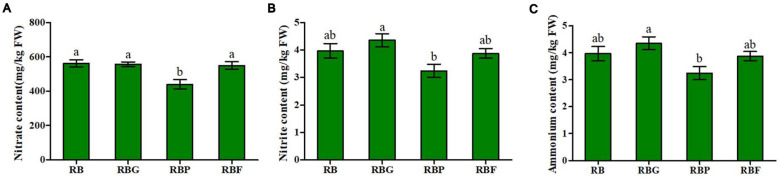
Effects of spectra on nitrate **(A)**, nitrite **(B)**, and ammonium content **(C)** in lettuce leaves. RB, 75% red + 25% blue LED; RBG, 60% red + 20% blue + 20% green LED; RBP, 60% red + 20% blue + 20% purple LED; RBF, 60% red + 20% blue + 20% infrared LED. The letters represent the significant difference of different treatments (*p* < 0.05).

The content of nitrate, nitrite, and ammonium in the lettuce shoots were shown in Figure 3. A significant decrease in nitrate content was determined under the RBP treatment compared with RB treatment. No significant difference was noted among the RB, RBG, and RBF treatments (Figure 3A). Similarly, ammonium content decreased by 18% under the RBP treatment relative to that under the RB treatment. A slight increase was determined under the RBG treatment relative to RB treatment (Figure 3C). As for the nitrite content change of the lettuce leaves under RBG, RBP, and RBF light, it did not reach a significant level relative to that under the RB light (Figure 3B). The RBG treatment exerted a positive effect on the increase of nitrite content in lettuce leaves, achieving a 9.8% increase relative to that under RB light, while about 18.4% decrease under RBP light.

As shown in Table 2, 16 types of amino acids were analyzed by the amino acid analyzer. Overall, the contents of Asp, Glu, and Leu in lettuce leaves were relatively high, accounting for more than 35% of the total free amino acids identified. The Met content was the lowest, accounting for only approximately 1% of the total number of amino acids. Relative to those under RB light, the contents of the 16 types of amino acids under RBG and RBP light sources increased to varying degrees, thereby causing the total free amino acid content of lettuce to increase by 19.9 and 25.1%, respectively. The content of Glu under RBG, RBP, and RBF light significantly increased by 31.8, 27.1, and 23.7%, respectively, relative to that under RB light. In particular, the RBG light source resulted in the highest Glu content.

**TABLE 2:** Effects of spectra on content (mg/g DW) of free amino acids in lettuce leaves.

**Amino acids names**	**RB**	**RBG**	**RBP**	**RBF**
Asp	1.575 ± 0.014c	1.980 ± 0.006a	1.970 ± 0.010a	1.697 ± 0.007b
Thr	0.720 ± 0.005c	0.830 ± 0.006b	0.897 ± 0.003a	0.717 ± 0.007c
Ser	0.455 ± 0.009d	0.537 ± 0.003b	0.590 ± 0.006a	0.487 ± 0.007c
Glu	1.800 ± 0.006d	2.373 ± 0.007a	2.287 ± 0.009b	2.227 ± 0.012c
Gly	0.880 ± 0.017c	1.027 ± 0.003b	1.100 ± 0.006a	0.887 ± 0.007c
Ala	0.970 ± 0.003c	1.147 ± 0.003b	1.210 ± 0.010a	0.977 ± 0.007c
Val	1.165 ± 0.009c	1.313 ± 0.003b	1.383 ± 0.003a	1.117 ± 0.007d
Met	0.165 ± 0.014a	0.173 ± 0.013a	0.180 ± 0.003a	0.110 ± 0.003b
Ile	0.895 ± 0.009c	1.053 ± 0.003b	1.110 ± 0.006a	0.887 ± 0.007c
Leu	1.475 ± 0.014c	1.710 ± 0.006b	1.820 ± 0.010a	1.450 ± 0.010c
Tyr	0.535 ± 0.003c	0.627 ± 0.012b	0.677 ± 0.009a	0.470 ± 0.006d
Phe	1.015 ± 0.014c	1.190 ± 0.003b	1.257 ± 0.009a	1.000 ± 0.006c
Lys	1.060 ± 0.035c	1.283 ± 0.012b	1.377 ± 0.007a	1.097 ± 0.007c
His	0.340 ± 0.006b	0.420 ± 0.003a	0.430 ± 0.006a	0.343 ± 0.003b
Arg	0.835 ± 0.003d	1.017 ± 0.007b	1.093 ± 0.003a	0.853 ± 0.003c
Pro	0.820 ± 0.006c	0.950 ± 0.006b	1.017 ± 0.003a	0.830 ± 0.006c
Free amino acids	14.705 ± 0.101d	17.630 ± 0.061b	18.397 ± 0.092a	15.147 ± 0.091c

*RB, 75% red + 25% blue LED; RBG, 60% red + 20% blue + 20% green LED; RBP, 60% red + 20% blue + 20% purple LED; RBF, 60% red + 20% blue + 20% infrared LED; Asp, aspartic acid; Thr, threonine; Ser, serine; Glu, glutamic acid; Gly, glycine; Ala, alanine; Val, valine; Met, methionine; Ile, isoleucine; Leu, leucine; Tyr, tyrosine; Phe, phenylalanine; Lys, lysine; His, histidine; Arg, L-arginine; Pro, proline.*

*The letters represent the significant difference of different treatments (*p* < 0.05).*

### Effect of LED Illumination Spectra on Enzyme Activity of Nitrogen Metabolism

**FIGURE 4:**
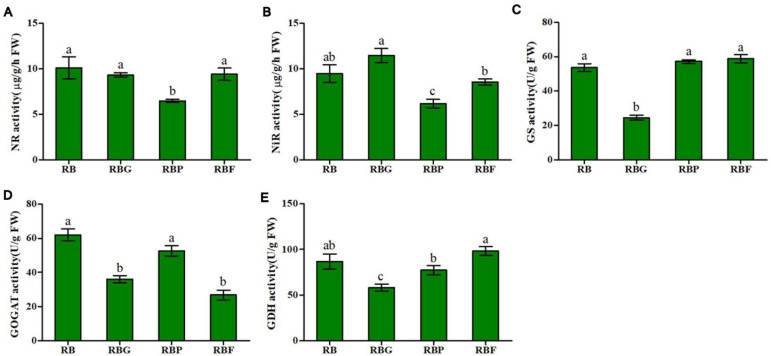
Effects of spectra on enzyme activity of nitrogen metabolism in lettuce. **(A)** NR activity, **(B)** NiR activity, **(C)** GS activity, **(D)** GOGAT activity, **(E)** GDH activity. RB, 75% red + 25% blue LED; RBG, 60% red + 20% blue + 20% green LED; RBP, 60% red + 20% blue + 20% purple LED; RBF, 60% red + 20% blue + 20% infrared LED. The letters represent the significant difference of different treatments (*p* < 0.05).

The activities of NR, NIR, GS, GOGAT, and GDH in lettuce leaves can be significantly affected by light quality. As shown in Figure 4A, relative to the activity of NR in lettuce under the RB light, that under the RBP light significantly decreased by 35.8%, whereas no significant changes were observed under the RBG and RBF treatments. A similar result was observed in NiR activity, which decreased by 34.8% under RBF light relative to that in the RB treatment (Figure 4B). As indicated by the results in Figures 4C–E, the quality of RBG light quality showed an inhibition effect on GS, GOGAT, and GDH activities relative to the quality of RB light, significantly decreasing them by 54.3, 41.9, and 32.9%, respectively. Relative to RB light, RBP light inhibited GDH activity, and RBF light significantly inhibited GOGAT activity, resulting in decreases of 10.9 and 15.2%, respectively.

### Effect of LED Illumination Spectra on Genes Expression of Light-Response and Nitrogen Metabolism

**FIGURE 5:**
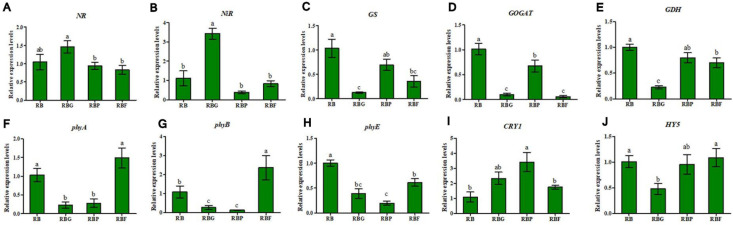
Effects of spectra on genes relative expression level of light-response and nitrogen metabolism in lettuce leaves. **(A)** Relative expression level of *NR*. **(B)** Relative expression level of *NIR*. **(C)** Relative expression level of *GS*. **(D)** Relative expression level of *GOGAT*. **(E)** Relative expression level of *GDH*. **(F)** Relative expression level of *phyA*. **(G)** Relative expression level of *phyB*. **(H)** Relative expression level of *phyE*. **(I)** Relative expression level of *CRY1*. **(J)** Relative expression level of *HY5*. RB, 75% red + 25% blue LED; RBG, 60% red + 20% blue + 20% green LED; RBP, 60% red + 20% blue + 20% purple LED; RBF, 60% red + 20% blue + 20% infrared LED. The letters represent the significant difference of different treatments (*p* < 0.05).

As shown in Figures 5A,B, different LED light qualities will significantly affect the expression level of *NR* and *NiR*. Herein, no significant upregulation or downregulation of *NR* expression was observed under the RBG, RBP, and RBF treatments relative to the RB treatment. Relative to that under RB light, *NR* expression was slightly upregulated (an increase by 40%) under RBG light. The expression of *NiR* in lettuce leaves under the RBG treatment was significantly upregulated, exceeding that under the RB light by 3.07 times. The expression of *NiR* under the RBP and RBF treatment showed no significant difference from that under the RB treatment. The expressions of *GS*, *GOGAT*, and *GDH* under the RBG and RBF treatments were significantly downregulated relative to those in the RB treatment (Figures 5C–E). Relative to the quality of RB light, the quality of RBP light significantly downregulated *GOGAT* expression and had no significant effect on *GS* and *GDH* expressions.

**FIGURE 6:**
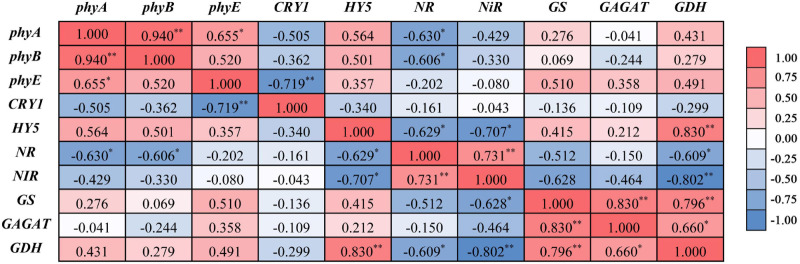
Correlation analysis of relative expression levels between light-response genes and nitrogen metabolism genes. **Correlation is significant at the 0.01 level (2-tailed), *Correlation is significant at the 0.05 level (2-tailed).

The relative expression levels of light-response genes are shown in Figures 5F–J. Relative to the RB light treatment, the RBG and RBP light treatments induced significant *phyA*, *phyB*, and *phyE* down-regulation and *CRY1* upregulation. The RBF light treatment had no significant effect on the expressions of *phyA* and *CRY1*, but is significantly up-regulation on *phyB* expression and down-regulation on *phyE* expression. Relative to RB light, RBG light significantly downregulated *HY5* expression whereas RBP and RBF light did not show significant effects. Correlation analysis showed that the relative expression levels between *phyA*, *phyB*, *HY5*, and NR had a significantly negative correlation. Moreover, a significantly negative correlation existed between *HY5* and *NR* expression (Figure 6). By contrast, a significant positive correlation was observed between the *HY5* and *GDH* expressions.

### Principal Component Analysis and Comprehensive Evaluation

**FIGURE 7:**
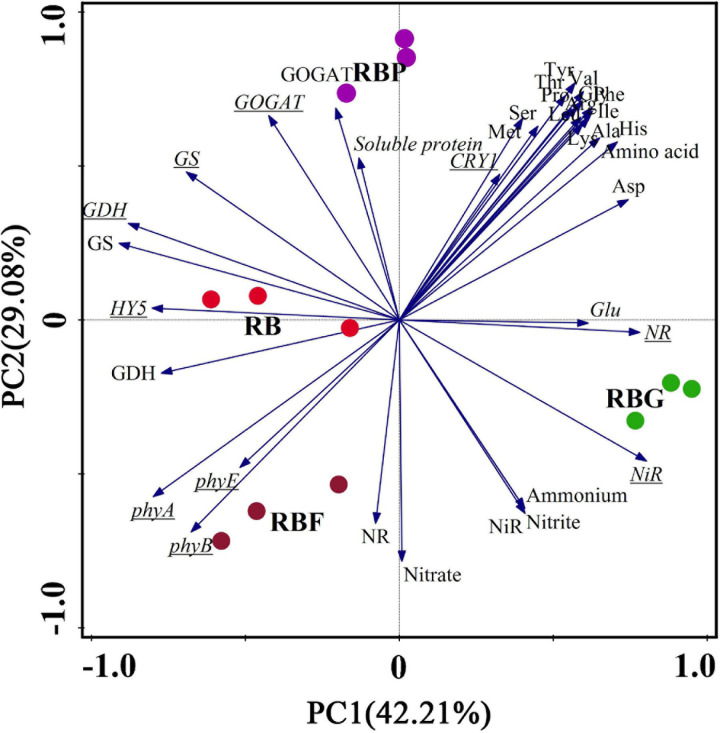
Principal component analysis (PCA) showing differences and correlations in nitrogen assimilation in lettuce leaves under different illumination spectrum. Scatter plot with different color indicated four light treatments (*n* = 3 replications). Underlined letters indicate genes expression. RB, 75% red + 25% blue LED; RBG, 60% red + 20% blue + 20% green LED; RBP, 60% red + 20% blue + 20% purple LED; RBF, 60% red + 20% blue + 20% infrared LED.

Principal component analysis was used to analyze 36 indexes of lettuce nitrogen metabolism under the four treatments (Figure 7). According to the results of the PCA, the nitrate and nitrite contents of lettuce leaves were closely correlated with ammonium content, and NR, NiR activities. Meanwhile, the following significant differences were observed: amino acid had a stronger intercorrelation with RBG and RBP light, and Glu showed the closest connection with RBG. NR and NiR activities and nitrate, nitrite, and ammonium content were closely correlated with RBF and RBG light and negatively correlated with RBP light. The expression of *HY5* was closely related to RB treatment and negatively correlated with the expressions of *NR* and *NiR*. The comprehensive effect of light quality on nitrogen metabolism in lettuce leaves can be evaluated by a scatter plot with different colors.

## Discussion

### Adding Green, Purple, and Far-Red Light Had Negative Effect on Lettuce Growth

Light is one of the most important environmental factors in regulating plant growth and development by serving as an energy source for plant photosynthesis and a regulator of plant physiology (Bian et al., 2018; Zhang et al., 2018). Previous studies have shown that the combination of red (600–700 nm) and blue light (400–500 nm) is an effective lighting source for crop growth by promoting metabolism and carbohydrates, including soluble sugars (Chen et al., 2016; Amoozgar et al., 2017). In our study, the biomass of aboveground lettuce was significantly higher under RB light than that under the other light treatments, especially RBG light (Figure 2). Similar results were observed for the soluble sugar content of lettuce leaves (Table 1). In other words, green, purple, and far-red light exert a negative effect on photosynthetic products and plant synthesis and growth. For plants, chlorophyll predominantly absorbs light in the red and blue portions of the spectrum and not in the green, purple, or far-red portions. When other light quality (green, purple, and far-red) is added to the combination of red and blue light, the effective red or blue light intensity decreases. In this study, the photon flux density of red and blue light under RBG, RBP, and RBF light was only 80% of that in the RB treatment. On the one hand, red and blue light intensity decreased after the addition of other light quality. On the other hand, photosynthesis might be inhibited under green, purple, and far-red illumination. Interestingly, the chlorophyll a content of lettuce showed a more significant decrease under RBG light than under RB light. Meanwhile, the chlorophyll content of lettuce increased more significantly under RBP or RBF light than under RB light (Table 1). The chlorophyll content caused the decrease of the photosynthetic rate and might explained why the FW and DW of above-ground of lettuce under RBG light were significantly lower than those in the RB treatment. However, the decrease of biomass under RBP or RBF light is difficult to explain in terms of chlorophyll content.

### Adding Purple Is Conducive to Vc Accumulation in Lettuce Leaves

For plants, light is not only the driving force for photosynthesis but also the transduction signal for regulating quality features, such Vc content. Previous studies have shown that blue or purple light could increase Vc content in lettuce (Chen et al., 2011; Zha et al., 2020). Consistent with previous results, our results indicate that the addition of purple light is conducive to Vc accumulation in lettuce leaves (Table 1). Compare with long-wavelength light (e.g., red or far-red light), short-wavelength light (e.g., blue, purple, or UV light) had higher energy at the same photon flux density. Under short-wavelength light, the antioxidant activity in plants is activated for adaption to increase photooxidative pressure (Gill and Tuteja, 2010; Wen et al., 2019). As a result, ascorbate, the reduced form of Vc, is synthesized by upregulating the gene expression and activity of ascorbate regeneration enzymes for cleaning up excessive reactive oxygen species (Zha et al., 2020). Thus, Vc accumulation induced by adding purple light might protect lettuce leaves form photooxidative pressure.

### Effect of Adding Green and Purple Light on Nitrogen Reduction

Nitrate is the main nitrogen source for plant growth and development, and it accumulates in the vacuoles of cells to act as an osmotic adjustment substances. A large amount of nitrate accumulates in cells when nitrogen is sufficient. Nitrate is reduced to nitrite by NR and reduced to ammonium by NiR. Ammonium is assimilated into organic nitrogen through the GOGAT/GS pathway. In the process, the accumulation of nitrogen metabolism intermediates exerts a direct impact on genes expression or enzyme activity, and it may significantly affect nitrogen metabolism (Perrin et al., 2016). In this study, the addition of purple light inhibited NR and NiR activities and caused relative lower nitrate, nitrite, and ammonium content (Figure 3). The possible reason is that higher level amino acids was accumulated in lettuce leaves and resulted in a feedback regulation in the absorption and reduction of nitrate through the inhibition of NR and NiR activities. In additional, adding green light caused an increase in nitrite content (Figure 3B). Relative to nitrate, excessive nitrite could be harmful to plants. To prevent excessive nitrite accumulation in leaves, plants possibly activate a protection mechanism by increasing *NiR* expression and activity (Figures 4B, 5B), and accelerated nitrite reduction, which leads to the increase in ammonium content under the RBG treatment in this work (Figure 3C). Bian et al. (2018) reported that adding green light to continuous red and blue light significantly reduces the nitrate content of lettuce leaves under continuous red and blue light. However, in the current work, nitrate content did not decrease significantly after the addition of green light (Figure 3A). Previous studies have shown that nitrate content is negatively correlated with the soluble carbohydrates in plants (Bouly et al., 2007). Nitrate plays a complementary role with soluble carbohydrates in maintaining cell osmotic pressure (Dapoigny et al., 2000). In this study, the addition of green light decreased the photosynthetic capacity of lettuce leaves and resulted in a relatively low soluble sugar content (Table 1). Hence, lettuce may increase the absorption of nitrate to maintain normal osmotic pressure.

### Adding Green, Purple, and Far-Red Light Had Contribution on Amino Acid Accumulation

In plants, the reduction of nitrate produces ammonium, which is then assimilated rapidly into organic nitrogen through the GS/GOGAT cycle and GDH pathway. GS has two isoenzymes, which are located in the cytoplasm and chloroplast. Synthesizing ammonium in chloroplast into glutamine is the main function of GS in chloroplast. Glutamine is synthesized under the action of GOGAT to complete the assimilation of ammonium. In this process, light quality also plays an extremely important regulatory role in enzyme activity and gene expression (Meya and Kowallik, 1995; Ning et al., 2019). The data in the current work demonstrated the positive effect of adding green, purple, and far-red light had contribution on amino acid accumulation (Table 2). This finding agrees with the results of previous studies (Sun et al., 2016; Wang et al., 2017; Zhang et al., 2018). Interestingly, relative to the RB treatment, the addition of green, purple, and far-red light caused a decrease in GS, GOGAT, and GSH activities and an increase in amino acid content. Moreover, the soluble protein content was lower under the RBG, RBP, and RBF treatments than under the RB treatment (Table 1). The speculation was that adding green, purple, and far-red light might inhibit protein biosynthesis as the precursor of amino acid and thereby cause the accumulation of amino acids.

### Relationship Between HY5 Gene and Nitrogen Metabolism

Plants sense light through specific photoreceptors and by monitoring environmental characteristics; hence, their sophisticated mechanisms have been evolved to determine light availability and quality (Jones, 2018). In general, phytochromes and cryptochromes respond to light condition (light quality, density, and photoperiod) and induced photomorphogenesis or metabolism through the HY5 transcription factor (Osterlund et al., 2000; Huang et al., 2013). In the presence of red or blue light, the COP1/SPA complex is inactivated by phytochromes or cryptochromes (Yi and Deng, 2005; Lau and Deng, 2012), leading to the accumulation of HY5 and the induction of photomorphogenesis (Laubinger et al., 2004). Signore et al. (2020) pointed out that the red spectrum induces phytochrome phototransformation and results in an increased NR activity. In the current work, the correlation analysis showed that the relative expression levels between *HY5* and *NR/NiR* had a significantly negative correlation (Figure 6). The speculation was that green light increased the nitrite and ammonium content because of the high NR and NiR activity and expression, which was negatively regulated by *HY5* (Figure 5). *HY5* might be an inhibitory factor of *NR* or *NiR* expression. The inhibitory effect was weakened when *HY5* was downregulated. In additional, *CRY1* was upregulated with the addition of purple light, which did not cause a significant change in downstream *HY5* (Figure 5). This finding agrees with the results of previous work, that is, *phy* or *CRY* upregulation inactivates the COP1/SPA complex, and *HY5* plays a regulatory role in downstream metabolize. In sum, *HY5* is possibly associated with *NR* and *NiR* expression and exerts a negative effect on nitrogen reduction. Therefore, further studies on the identification of HY5 functions for nitrate assimilation via Y1H assay or genome editing should be valuable.

## Conclusion

Our results indicated that LED illumination spectra exerted a significant effect on the growth and nitrogen metabolism of lettuce. Adding green, purple, and far-red light had a negative effect on lettuce growth because of the decrease in the effective photon flux density for chlorophyll absorption. However, purple was found to be conducive to Vc accumulation in lettuce leaves. For nitrate reduction, adding green light caused an increase in nitrite and promoted the reduction from nitrite to ammonium through the activation of *NiR* expression and enzyme activity. On the contrary, adding purple light inhibited NR and NiR activities and caused a low nitrate, nitrite, and ammonium content. For nitrogen assimilation, the addition of green and purple light contributed to amino acid accumulation. Transcription factor HY5 might mediate the regulation of light quality on nitrogen metabolism by inhibiting *NR* and *NiR* expression, and it might have a negative effect on nitrate reduction. The lettuce under RBP treatment had higher comprehensive properties than other light, which could be used for reference in supplemental lighting strategy greenhouse production. Further studies on the identification of *HY5* functions for nitrate assimilation via genome editing techniques will be valuable.

## Data Availability Statement

The original contributions presented in the study are included in the article/Supplementary Material, further inquiries can be directed to the corresponding authors.

## Author Contributions

JW was the recipient of funds. TW, KH, and ML conceived the experiment. JL, ML, and YL prepared the plant materials, collected samples, and undertook experiments. JL analyzed the data and prepared the manuscript. All authors contributed to the manuscript revision.

## Conflict of Interest

The authors declare that the research was conducted in the absence of any commercial or financial relationships that could be construed as a potential conflict of interest.
