# Supplementary material for: Effect of LED Spectrum on the Quality and Nitrogen Metabolism of Lettuce Under Recycled Hydroponics
Source: Front Plant Sci. 2021 Jun 17;12:678197. doi: 10.3389/fpls.2021.678197 (PMC8247776; doi:10.3389/fpls.2021.678197)
Supplement: Supplementary file 1 [file Data_Sheet_1.docx]

Table S1 The Garden test formula of nutrient solution (Unit: g/1000L) (Cited from Hori, 1966)

| Macronutrient fertilizer |  | Trace element fertilizer | | |  | Trace element fertilizer |  |
| --- | --- | --- | --- | --- | --- | --- | --- |
| Ca(NO_3_)·4H_2_O | 950 | | NaFe-EDTA | 20 | | CuSO_4_·5H_2_O | 0.05 |
| KNO_3_ | 810 | | H_3_BO_3_ | 3 | | (NH_4_)_6_Mo_7_O_24_·4H_2_O | 0.02 |
| MgSO_4_·7H_2_O | 500 | | MnSO_4_·4H_2_O | 2 | |  |  |
| NH_4_H_2_PO_4_ | 155 | | ZnSO_4_·7H_2_O | 0.22 | |  |  |

Table S2 The primer sequences of primers for qRT-PCR

| Gene | Primer Sequence（F） | Primer Sequence（R） |
| --- | --- | --- |
| Actin | AGGTGTCATGGTTGGCATGGGA | TGTTCTTCAGGGGCGACACG |
| *NR* | GGAGGTGGGAAGAAAGTGACA | TAGCGACCAAAAACACCAACA |
| *NiR* | CACCACACGTCAAAACTGGC | GAGTGGATTGCCAACTGGGC |
| *GS* | ATGGCTAAGACAAAGAGTATGAC | AAGTACCACATATCTTGCGTAG |
| *GOGAT* | CAGGGAAACTTAAACTGGATGC | TTTATGGCACTTGTAGCCGTAT |
| *GDH* | CTTTAGCAGCAACAAATCGTAAC | GGCTTCCCAACCTCTTAGAA |
| *phyA* | CTTCACACTAGTGCTTTGTGTC | ACTTGACTTATACATGCACCCA |
| *phyB* | CCATGCTCAATACATGGCTAAC | TAACGTAATGGAAACGGAATGC |
| *phyE* | CCTCAAAGATGAAGACACGTTG | ATCTTCACCAAAAGACACTTGC |
| *CRY1* | CGATTACAAGAAGCGCTTACTC | TAGGGTTTGTTCTCAGCGTATT |
| *HY5* | CGGCTAGTCTACCTTCAAGTAG | CGTCATCACTTTCCATTCCTTC |
